# Eco Friendly Approach for Synthesis, Characterization and Biological Activities of Milk Protein Stabilized Silver Nanoparticles

**DOI:** 10.3390/polym12061418

**Published:** 2020-06-24

**Authors:** Sadanand Pandey, Corli De Klerk, Joonwoo Kim, Misook Kang, Elvis Fosso-Kankeu

**Affiliations:** 1 Particulate Matter Research Center, Research Institute of Industrial Science & Technology (RIST), 187-12, Geumho-ro, Gwangyang-si, Jeollanam-do 57801, Korea; realjoon@rist.re.kr; 2 Department of Chemistry, College of Natural Science, Yeungnam University, 280 Daehak-Ro, Gyeongsan, Gyeongbuk 38541, Korea; 3 Water Pollution Monitoring and Remediation Initiatives Research Group, School of Chemical and Minerals Engineering, North-West University, Potchefstroom 2531, South Africa; corlideklerk@gmail.com (C.D.K.); Elvis.FossoKankeu@nwu.ac.za (E.F.-K.)

**Keywords:** nanotechnology, biopolymer, milk protein, silver nanoparticles, antimicrobial activity

## Abstract

Today, the overall occurrence of re-emerging and rising illnesses has been a serious load on economies as well as public health. Here, we describe a simple, nontoxic and eco-friendly method for the synthesis of milk protein (MP)-stabilized silver nanoparticles (MP-s-AgNPs) using ultrahigh-temperature full cream milk. Highly stable AgNPs were prepared with a fair control over their size, without using any reducing or stabilizing agent, and their formation was attributed to the presence of the MP casein. Ag^+^ ion reduction was possibly caused by the MPs. The synthesized MP-s-AgNPs were characterized in detail by ultraviolet-visible spectroscopy, Fourier-transform infrared spectroscopy, X-ray diffraction, scanning electron microscopy, transmission electron microscopy and dynamic light scattering. MP-s-AgNPs showed inhibitory activity against both Gram-positive (*Bacillus subtilis* and *Staphylococcus aureus*) and Gram-negative microorganisms (*Salmonella typhi* and *Escherichia coli*). Moreover, MP-s-AgNPs were found to be more toxic to bacteria than to fungi (*Aspergillus fumigatus*, *Aspergillus ochraceus* and *Penicillium chrysogenum).*

## 1. Introduction

Nanotechnology constitutes an impressive power tool in order to check out the overall darkest alternatives in reference to medical sciences in numerous ways such as imaging [1], catalysis [2], sensing [3,4,5], targeted drug delivery [6], gene delivery [7] and artificial implants [8]. Currently, silver nanoparticles (AgNPs) are extensively used in therapeutic, pharmaceutical and agricultural industries, water processing and antimicrobial applications against a wide range of bacteria and fungi.

AgNPs are used as deodorizers and disinfectants [9,10] by incorporating them into garments, bandages, coatings and food containers; in addition, they are used to purify drinking water, and recently, as an insecticide to control the attack of pests on various crops [11]. Most of these applications of AgNPs are attributed to their size- and shape-dependent unique chemical and physical characteristics [12,13,14]. Nanoparticles are usually synthesized by chemical or biological methods [15,16,17,18]. AgNPs have been synthesized by borohydride reduction [19], acrylate/citrate reduction [20,21], polyol process [22,23], microwave irradiation [24,25], plant extract and vegetable oil-based process [26,27], photoreduction [28], amino acid-based process [29], etc. However, the chemical synthesis of AgNPs is elaborate and causes adverse effects on the ecosystem. On the other hand it was well known that the biological materials have the great potential to reduce the metal ions into metal NPs. Biosynthesis of AgNPs gained considerable attention in the past decade. Moreover, it has been proposed as a less toxic, cost-effective, environmentally friendly alternative to chemical and physical methods. Accordingly, AgNPs have been synthesized using microorganisms, plant extracts and animal milk [30,31,32,33,34,35,36,37,38]. Mubarak et al. [39] demonstrated the green synthesis of AgNPs using *Oscillatoria willei* NTDM01. Patel et al. [40] reported the synthesis of AgNPs using Cyanobacterial extracts like polysaccharides and phycocyanin. Synthesis of NPs using different animal milk like sheep milk, camel milk and goat milk was earlier reported by a few researchers. Basically, the proteins present in the biological materials are involved in the reduction and stabilization of NPs [41,42,43]. However, until now, the use of proteins to synthesize AgNPs has not been much explored.

Milk is an economical and readily available protein source. Milk contains 3.3% total protein. Milk proteins (MPs) contain all nine essential amino acids required by humans. In bovine milk, approximately 82% of MP is casein and the remaining 18% is serum or whey protein. Casein is stable to heat treatment. Therefore, the normal high-temperature short time pasteurization conditions do not influence the functional and nutritional properties of casein. However, high-temperature treatment can cause interactions between casein and whey protein, which can affect the overall functional properties, but not the nutritional properties [44,45,46]. In this study, we used milk to synthesize AgNPs because milk does not contain any toxic material, is environmentally safe and does not release any harmful chemicals into the environment; moreover, milk is a renewable resource, with unlimited supply and easily availability. Biotolerable and highly stable MP-stabilized silver nanoparticles (MP-s-AgNPs) were prepared with a reasonable control over their size, without utilizing any reducing or stabilizing agent. The proteins present in Clover milk were possibly responsible for Ag^+^ ion reduction. The synthesized MP-s-AgNPs were thoroughly characterized by ultraviolet-visible (UV-Vis) spectroscopy, Fourier-transform infrared (FTIR) spectroscopy, X-ray diffraction (XRD), scanning electron microscopy (SEM) and transmission electron microscopy (TEM). In addition, the overall antimicrobial activity of MP-s-AgNPs was tested toward Gram (+ve) (*Bacillus subtilis* and *Staphylococcus aureus*) and Gram (−ve) bacteria (*Salmonella typhi* and *Escherichia coli*) and fungi (*Aspergillus fumigatus*, *Aspergillus ochraceus* and *Penicillium chrysogenum*).

## 2. Materials and Methods 

### 2.1. Chemicals and Cultures 

Commercial ultrahigh-temperature (UHT) full cream milk (brand: Clover; 250 mL) was purchased from Pick n Pay Stores (Potchefstroom, South Africa). The principal constituents of Clover full cream milk (unit size: 1 L; barcode: 6001299002343) contain Protein (8 g), glycemic carbohydrates (12 g), total fat (8.5 g), sodium (123 mg), vitamin B12 (1.0 μg), vitamin B2 (0.4 mg), calcium (293 mg) and phosphate (218 mg) per 250 mL. Luria Bertani (LB) nutrient broth (10 g/L tryptone, 5 g/L yeast extract and 10 g/L NaCl) was purchased from (Sigma–Aldrich, Saint Louis, MO, USA)Agar powder (318078) and silver nitrate (≥99.0) were purchased from (Sigma Aldrich, cat. 209139, St. Louis, MO, USA). All chemicals were used without further purification. Ultrapure water with a resistivity of 18.2 MΩ.cm was utilized as the solvent in all preparations.

### 2.2. Biosynthesis of Silver Nanoparticles from Commercial Milk

**Figure 1:**
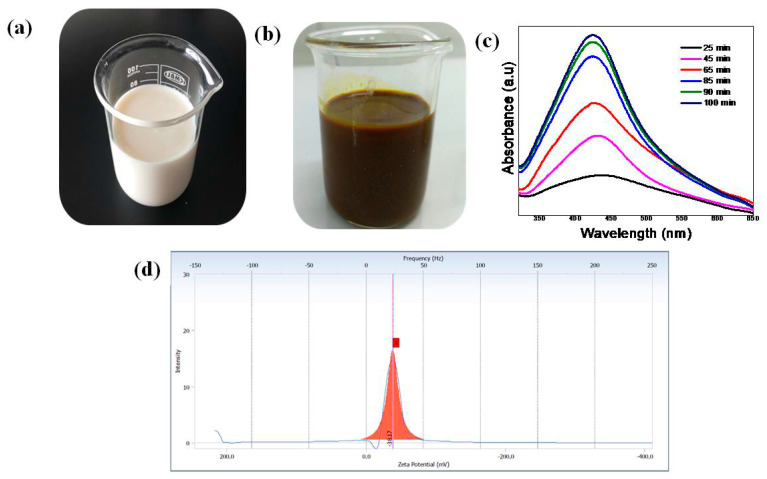
Photographs of (**a**) commercial milk and (**b**) milk protein-stabilized silver nanoparticles (MP-s-AgNPs) solution after 100 min and (**c**) UV-Vis spectra of MP-s-AgNPs recorded at different time interval. (**d**) Zeta potential plot for AgNPs produced using milk, showing the excellent stability of MP-s-AgNPs.

Biosynthesis of AgNPs was carried out as described by Pandey et al. (2012) [3]. Silver nitrate solution (80 mL; 1 mM) was taken in an Erlenmeyer flask. Then, 20 mL of milk was added to AgNO_3_ solution under vigorous stirring at 60 °C. The color of the reaction mixture gradually changed from milky white to light yellow, and finally, to dark brown in 90 min. The reaction was allowed to proceed for 90 min to ensure that the reaction was complete, which resulted in the formation of a MP-s-AgNP suspension. This suspension was then placed in the dark at room temperature (RT) to limit the photo-activation of silver nitrate. Reduction of Ag^+^ to Ag^0^ was confirmed from the color change of the solution from milky white to brown as shown in Figure 1a,b. The reaction product was then separated by centrifugation at 10,000 rpm for 30 min and purified by redispersion of the pellet in deionized water (DI water). Biosynthesized MP-s-AgNPs were finally collected by centrifugation at 10,000 rpm for 30 min, washed twice with DI water and unbound proteins removed by treating with 80% (v/v) ethanol. The purified nanoparticles solutions were frozen, keeping at −80 °C for 30 min and then immediately shifted into a freeze-dried device with a vacuum for 48 h, with the condenser surface temperature maintained at −80 °C throughout the experiments. After freeze-drying, the samples were reconstituted by adding Milli-Q water in the vial, and thereafter the sample was maintained at room temperature for 30 min to ensure proper cake wetting. Then, a gentle vortex was carried out for 30 min to ensure complete homogenization of the sample. The reconstituted sample was analyzed using UV-Vis spectrophotometer, surface charge analysis (zeta potential), SEM and TEM.

### 2.3. In Vitro Antimicrobial Activity of MP-s-AgNPs

#### 2.3.1. Antimicrobial Screening

The antimicrobial performance of the synthesized MP-s-AgNPs was tested against Gram (−ve) and Gram (+ve) bacteria; these were selected because they had different cell membrane characteristics. In addition, the antifungal activity of MP-s-AgNPs toward three common fungi: *Aspergillus fumigatus*, *Aspergillus ochraceus* and *Penicillium chrysogenum* was investigated. The antimicrobial activity of MP-s-AgNPs was compared to those of reference compounds. The agar disc diffusion method (qualitative) and broth dilution approach (quantitative) were used to determine the antimicrobial activity and effectiveness of the synthesized MP-s-AgNPs.

##### 2.3.1.1. Antibacterial Screening

Strains of Gram (−ve) (*Escherichia coli* and *Salmonella typhi*) and Gram (+ve) (*Staphylococcus aureus* and *Bacillus subtilis)* bacteria were selected to assess the potential antibacterial effects of the produced MP-s-AgNPs. The bacterial samples were prepared by sub-culturing each of the strains on LB agar plates (10 g/L tryptone, 5 g/L yeast extract and 10 g/L NaCl) incubated at 35 °C for 16 h. Typically, 2–3 single colonies were selected and suspended in a nutrient broth and used for further experiments.

• Disc Diffusion Method for Antimicrobial Susceptibility Testing

Bacterial sensitivity toward antibiotics is commonly tested by disc diffusion analysis. We conducted the analysis using discs laden with the synthesized MP-s-AgNPs. First, MP-s-AgNPs were dissolved in DI water at 50 °C and allowed to cool down to RT. Sterile blank diffusion discs (6 mm diameter, Davies Diagnostics (Pty) Ltd., South Africa) were infused with 15 µL with a certain concentration to obtain final disc concentrations of 800, 400, 200, 100, 50, 25, 12.5 and 6.25 µg/disc. The discs were air-dried for 0.5 h and carefully placed on the surface of 90 mm LB agar plates freshly inoculated with bacteria. Then, the discs were left undisturbed for 10 min at RT for settling and incubated in an inverted position for 24 h at 37 °C.

All antimicrobial tests were performed in quadruplicate (four discs using an identical concentration), and commercial antibiotics were used as positive controls: Vancomycin (30 µg/disc) for Gram (+ve) bacteria and carbenicillin (100 µg/disc) for Gram (+ve) bacteria. Clear zones surrounding the discs were identified as growth restriction zones. The susceptibility zone was determined as the average of replicate values of the inhibition zone diameter. The radius of the inhibition zone of bacterial growth was measured along two axes at right angles to each other. The minimum inhibition zone (*MIZ*) was determined using the following equation:

(1) $$\%\;Minimum\;Inhibition\;Zone\;\left(MIZ\right)=\frac{\pi r^{2}}{\pi R^{2}}x100$$

where *r* = *r*_2_ − *r*_1_; *r*_2_ is the radius of the inhibition zone generated by the control sample containing commercial antimicrobial agents, *r*_1_ is the radius of the inhibition zone generated by the prepared antimicrobial agent and *R* is the radius of the Petri dish.

• Broth Dilution Method for Antimicrobial Susceptibility Testing

Solutions of the tested MP-s-AgNPs were serially diluted two-fold in LB nutrient broth in sterile test tubes to obtain concentrations of 800, 400, 200, 100, 50, 25 and 12.5 µg/mL. Then, 2 mL of an overnight grown culture of each microorganism with a concentration of approximately 105 cells/mL was added to the test tubes to yield a final volume of 4 mL. Next, the mixtures were incubated at 37 °C for 18 h, and the growth was examined by UV-Vis absorbance measurements at 600 nm with an UV-Vis spectrophotometer. The minimum inhibitory concentration (MIC), which is the lowest concentration of MP-s-AgNPs required to inhibit the visible growth of organisms, was determined. The solution in each tube was serially diluted and incubated on agar plates for 24 h at 37 °C to determine the minimum bactericidal concentration (MBC), which is the highest dilution of the synthesized MP-s-AgNPs required to kill > 99.9% of the initial bacterial inoculum.

##### 2.3.1.2. Antifungal Screening

• Disc Diffusion Assay

Disc diffusion assay was performed to evaluate the antifungal capability of the synthesized MP-s-AgNPs. For the assay, *Aspergillus fumigatus*, *Aspergillus ochraceus* and *Penicillium chrysogenum* fungi were used. Mature conidia of the fungi were harvested and grown in Sabouraud dextrose nutrient broth. First, the MP-s-AgNPs were dissolved in pure DI water. Sterile 6-mm diffusion discs were prepared to obtain final disc concentrations of 800, 400, 200, 100, 50, 25, 12.5 and 6.25 µg/disc; then, the discs were air-dried and placed in Petri dishes, in which 1 mL of inoculated broth was evenly spread with sterile forceps. The experiments were conducted in triplicate with each disc impregnated with the same concentration of the antimicrobial compound. The plates were then incubated at 37 °C for 48 h. The reference compound used for antifungal screening was Amphotericin B (AmB; 20 µg/disc). The radius of the inhibition zone of fungal growth was measured along two axes at right angles to each other. The disc diffusion method used for antibacterial assay was applied for antifungal screening. 

### 2.4. Characterization of the Synthesized MP-s-AgNPs

Analysis of surface plasmon resonance was carried out with UV-vis spectrophotometer (Shimadzu model UV-1208, Shimadzu, Tokyo, Japan) operating in the 250–800 nm range. All samples solutions were diluted 1:10. The Zeta Potential of samples were evaluated using a Malvern Zetasizer ZS. 3600 (Malvern Instruments, Worcestershire, UK) FTIR spectra of clover milk (M) and MP-s-AgNPs were taken in KBr pellets using a Perkin Elmer 1600 FT-IR spectrophotometer (Perkin Elmer, Inc., MA, USA)in the range of 4000–400 cm^−1^. The powder X-ray diffraction patterns of the nanoparticles were performed by using X-ray diffractometer Ultima IV (Rigaku, Tokyo, Japan). Samples analyses were done employing Cu Kα radiation at the wavelength of 1.5406 Å with visible lights at 45 kV/40 mA. The surface morphology of the M and MP-s-AgNPs samples were examined by a scanning electron microscopy SEM (VEGA 3, TESCAN, Brno, Czech Republic) under a 20 kV electron acceleration voltage by carbon coating of samples. The presence of AgNPs within MP-s-AgNPs was confirmed by using (JEOL JEM-2100F, Tokyo, Japan), field emission electron transmission microscope (TEM) with an accelerating voltage of 90 kV. The particle size distribution of the AgNPs obtained from TEM images was evaluated using Image J 1.45 s software. Thermal gravimetric analysis (TGA) was carried out by using TGA (N-1000, SCINCO, Seoul, Korea) from 25 to 900 °C at a heating rate of 10 °C min^−1^ under nitrogen atmosphere.

## 3. Results and Discussion

### 3.1. Characterization of MP-s-AgNPs 

#### 3.1.1. UV-Vis Spectroscopic Analysis of MP-s-AgNPs

The formation of MP-s-AgNPs was investigated by UV-Vis spectroscopy. The white color of milk indicates that scattering is more dominant than absorption in the entire visible wavelength range. As a result, when light interacts with such particles, the angular distribution of the scattered radiation is governed by the Lorenz–Mie theory. In such cases, light does not equally scatter in all directions, but mostly in the forward direction. For most diluted solutions, although these effects are not visible, the recorded spectra are reliable. Thus, in this study, we diluted the MP-s-AgNPs in a cuvette before UV-Vis spectroscopy analysis. At zero minutes no SPR band was observed and the solution color was milky white. After reaction at 60 °C for 25 min, the Ag-NPs obtained showed a significant change in the UV-vis absorption peak, a characteristic SPR band for Ag-NPs, centered at 435 nm (Figure 1c). As shown in Figure 1c, the intensity of the SPR peak increased as the reaction time increased, which indicated the continued reduction of the silver ions, and the increase of the absorbance with the reaction time indicates that the concentration of Ag-NPs increases [47]. When the reaction time reached 65 min the absorbance was increased, and the λmax value was slightly blue-shifted to 429 nm. For reaction times of 85 and 90 min, the absorbance was also increased and blue-shifted to 426 and 424 nm, respectively. This phenomenon indicated that the size of particles was decreased because the absorbance peak due to the SPR of metal nanoparticles shows the blue-shift with decreasing particle size [48]. At the end of the reaction (100 min), the absorbance was considerably increased and there was no significant change in the λmax value (423 nm), compared with the 90 min reaction time. The SPR absorption of metal nanoparticles depends on the particle size and the dielectric constant of the medium. SPR patterns, characteristics of metal nanoparticles strongly depend on particle size, stabilizing molecules or the surface adsorbed particles and the dielectric constant of the medium [49,50,51,52]. The single SPR band in the early stages of synthesis corresponds to the absorption spectra of spherical nanoparticles. Similar findings have been also reported by other researchers [53,54]. Several researchers [55,56,57] considering SPR have reported similar observation. The UV-Vis peak at λmax = 423 nm clearly suggested the formation of spherical AgNPs [58], which was further confirmed by TEM analysis.

The AgNPs were found to be highly stable, and no significant change in the SPR band was observed even after several months. This indicated strong interaction of milk proteins, mainly casein, with AgNPs, which prevented the agglomeration of AgNPs induced by electrostatic imbalance. The AgNPs were lyophilized, stored as a dry powder and then, redispersed in aqueous media whenever required.

#### 3.1.2. Zeta Potential Analysis

Determination of Zeta potential (ζ) is crucial because it provides information about the nanoparticle surface states as well as the long-term stability of nanoparticles. The overall ζ of the MP-s-AgNPs was evaluated to determine their stability against aggregation. The absolute value of ζ is an indicator of the colloidal system stability. The ζ value of the MP-s-AgNPs was close to −38.17 mV shown in Figure 1d, indicating the high stability of the AgNPs, as reported in the literature [59,60,61,62,63,64]. The stability of MP-s-AgNPs could be attributed to the presence of molecules adsorbed on the surface of the nanoparticles during biosynthesis. This value indicates good stability of the AgNPs. The ζ value for the AgNPs produced in this study indicated good stability of the colloid, which could be attributed to the presence of MPs that functionalized the AgNPs. The solution had the highest negative value of the Zeta potential (−38.17 mV), and so was the most stable because the repulsive forces between negatively charged particles prevent agglomeration [64,65].

The stability of MP-s-AgNPs was studied for three months. No changes in ζ and particle size were observed for three months. The ζ value remained almost constant throughout the experimental period of three months. This confirmed the high stability of the synthesized AgNPs. In addition, we recorded the SPR band of the AgNPs stored at 4 °C for three months. The spectral features of freshly prepared MP-s-AgNPs and those stored for three months were the same, clearly indicating the long-term stability of the AgNPs. A similar finding was also reported in literature [66]. The biomolecules present in milk formed a protective layer on the nanoparticle surface, and strong physical adsorption of biomolecules onto the surface of the AgNPs led to enhanced stabilization. Thus, because of the nanosize of the particles and high stability, the synthesized MP-s-AgNPs showed excellent antibacterial activity [67,68,69].

It is the nature of the size that differs as measured by dynamic light scattering (DLS) and TEM. DLS provides larger average hydrodynamic diameter size due to the expansive nature of the organic matrix surrounding the nanoparticles, plus the liquid layer around the particle, which formed a larger hydration layer but size measured by TEM gives the actual size of the NP. Additionally, the size measured by DLS is estimation not the actual size of NPs [69]. Thus we have further characterized the AgNPs using TEM.

#### 3.1.3. TEM Analysis of MP-s-AgNPs 

**Figure 2:**
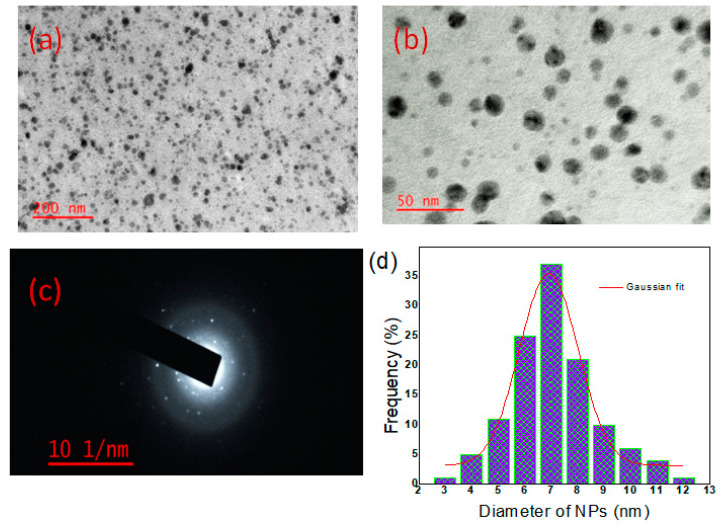
TEM images of AgNPs synthesized by commercial milk. (**a**) At lower magnification; (**b**) at higher magnification; (**c**) SAED pattern of the AgNPs showing concentric rings indicate the high crystallinity of the NPs and (**d**) particle size distribution histogram with a Gaussian curve fitting.

TEM analysis provided further insight into the morphology, size, as well as the size distribution of the synthesized AgNPs (Figure 2). The AgNPs produced by the reduction of Ag^+^ to Ag^0^ were spherical and had smooth surfaces. All the AgNPs were well separated, without any agglomeration. The XRD results were in good agreement with the selected area electron diffraction (SAED) pattern, which revealed that the AgNPs were polycrystalline in nature (Figure 2c) [3]. The TEM images (Figure 2a,b) showed that the MP-s-AgNPs were distinct, spherical and completely separated from each other. The sizes of more than 150 particles were measured. From the histogram, the particle size was found to be in the range of 3–12 nm (Figure 2d). The average particle size determined from the TEM images (Figure 2) was 7.0 nm. The use of MP-s-AgNPs of the 7.0 nm mean size may result in better antimicrobial activity.

#### 3.1.4. FTIR Spectroscopy and XRD Analyses of MP-s-AgNPs

FTIR spectroscopy is a valuable tool to study the secondary structural nature of proteins in various environments [70]. FTIR analysis was performed to identify the biomolecules in milk that were responsible for the reduction of Ag^+^ ions. It was found that secondary (2°) amines present in milk acted as capping agents, providing stability to the AgNPs.

**Figure 3:**
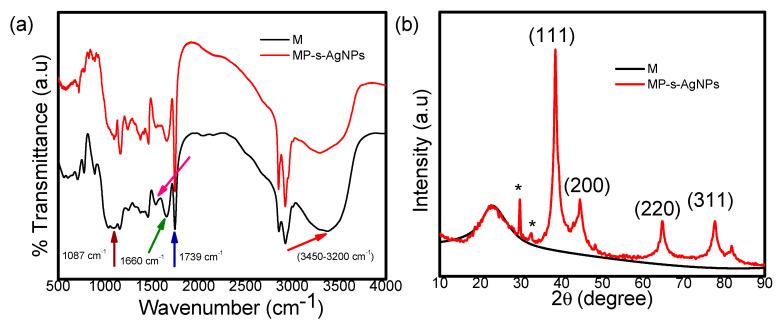
(**a**) FTIR spectra of commercial milk (M) and MP-s-AgNPs and (**b**) shows the X-ray diffraction pattern of commercial milk and MP-s-AgNPs.

The FTIR spectra of milk and MP-s-AgNPs in the 400–4000 cm^−1^ range are presented in Figure 3a. The FTIR spectrum of milk (Figure 3a) showed strong absorption peaks in the range of 3450–3200 cm^−1^, which were attributed to the hydroxyl group and H-bonded OH stretching vibrations [71,72]. In addition, the band at 1660 cm^−1^ indicated the disordered protein structure, which was attributed to a high content of proline residues (Figure 3a) [73,74]. MP-s-AgNPs exhibited peaks at 1553 cm^−1^ and 1087 cm^−1^, which were attributed to the interaction of AgNPs with the MPs (Figure 3a). A comparison of the FTIR spectra (Figure 3a) revealed the relative shifts in the peak positions and intensity distribution. This clearly showed that the MPs capped the surfaces of the nanoparticles, providing stability to the nanoparticles for long periods. Moreover, the characteristic C=O stretching band of the carboxylic acid group was observed at 1739 cm^−1^ and 1749 cm^−1^ in milk and MP-s-AgNPs sample respectively. The C–O stretching band was superimposed by the broad band centered at 1095 cm^−1^ corresponding to the C–O–C symmetric stretching and C–O–H bending vibrations of the MPs. The prominent bands around 1661 cm^−1^ and 1557 cm^−1^ could be attributed to amides I and II of proteins [75,76,77], respectively. The peak at 1600–1700 cm^−1^ was due to the carbonyl (C=O) stretching amide I [41]. The absorption at region 1510–1580 cm^−1^ corresponded to the N-H bending, contributed by the C–N stretching (amide II) and the phenyl nucleus (C=C) [73,75]. Thus, the proteins were bound to the AgNPs through free amine groups or the carboxylate ion of the amino acid residue. The C=O stretching band indicated that the –COOH group in the milk was bound to the AgNPs. Thus, the bands at 1739 cm^−1^ and 1087 cm^−1^ in IR indicate the possibility that AgNPs are bound to proteins through free amine groups. These compounds might have interacted with the Ag surface, making the Ag NPs highly stable. It is quite evident that proteins form a coat covering the AgNPs in order to prevent their agglomeration and enhance the stabilization in the medium. Previous researchers demonstrated the presence of proteins in the synthesized NPs through FTIR analyses [78,79,80,81]. It has been reported that proteins can bind to NPs either through free amine groups or cysteine residues in the proteins [79,82,83] and may possibly stabilize the Ag NPs. The plausible mechanism for the NPs biosynthesis and the role of proteins during the process have been reported well by Ahmad et al. [84] and Kalimuthu et al. [85] Apart from this few other earlier reports of the FTIR spectroscopic studies on Ag NPs obtained from the fungus, *Penicillium brevicompactum* WA 2315 and *Cladosporium cladosporioides*, are in agreement with our report [79,80]. Those studies confirmed that the carbonyl groups from the amino acid residues and peptides of proteins have a strong ability to bind to Ag. Therefore, from all the reported literatures, it was confirmed that the biomolecules could function in the development and stabilization of the Ag NPs in an aqueous medium.

The XRD pattern of milk showed a broad intense peak at 2θ = 22.9°, indicating its amorphous nature (Figure 3b). Figure 3b also displays the XRD pattern of the synthesized MP-s-AgNPs. As observed, the pattern contained four well-defined diffraction peaks at 38.6°, 44.4°, 64.7° and 77.6° corresponding to the (111), (200), (220) and (311) planes of the face-centered cubic (fcc) crystal structure of metallic silver, respectively. The interplanar spacing (d_hkl_) values (2.348, 2.030, 1.437 and 1.229 Å) calculated from the XRD pattern of the MP-s-AgNPs sample matched with those for standard silver (JCPDS PDF card No. 04–0783) [86,87,88]. All the reflections correspond to pure silver metal with fcc symmetry. The high intense peak for fcc materials is generally (1 1 1) reflection, which is observed in the MP-s-AgNPs. The intensity of peaks reflected the high degree of crystallinity of the AgNPs. Few unassigned XRD peaks around 29 and 32 degree were also observed suggesting that the crystallization of bio-organic phase occurs on the surface of the AgNPs. Similar results were also reported in synthesis of AgNPs using edible mushroom extract, geranium and *Coleus aromaticus* leaf extract [89,90,91].

#### 3.1.5. SEM Image Analysis of MP-s-AgNPs

**Figure 4:**
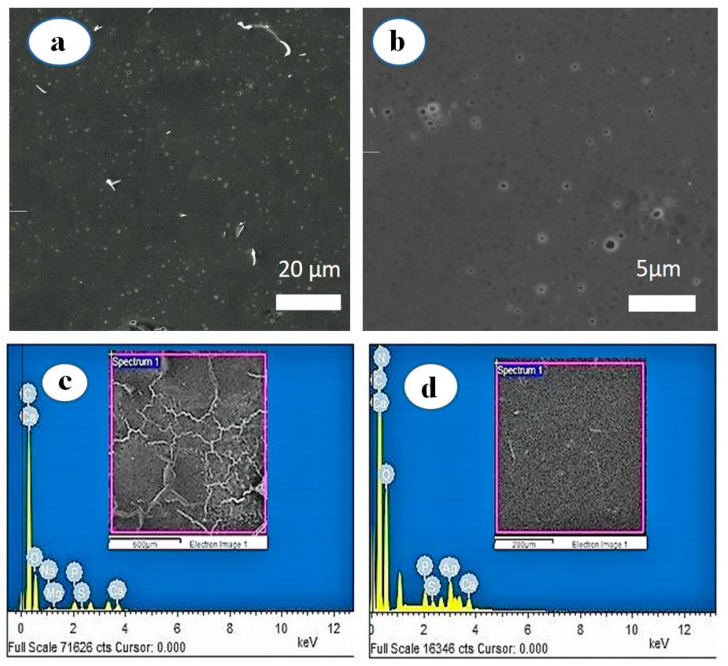
SEM images of AgNPs synthesized by commercial milk. (**a**) At lower magnification and (**b**) at higher magnification and EDS profiles of (**c**) commercial milk and (**d**) MP-s-AgNPs.

The low-and high-magnification SEM images of the synthesized MP-s-AgNPs are presented in Figure 4a,b, illustrating relatively spherical and uniform AgNPs. The SEM image of AgNPs was due to interactions of the hydrogen bond and electrostatic interactions between the milk macromolecule capping molecules bound to the AgNPs. The AgNPs were not in direct contact, even in the aggregates, indicating that the AgNPs were stabilized by the capping agents.

#### 3.1.6. Energy Dispersive Spectroscopy (EDS) Analysis of MP-s-AgNPs

The SEM-EDS profiles of milk and MP-s-AgNPs are given in Figure 4c,d, respectively. As observed, the Ag elemental peak was observed only for MP-s-AgNPs and not for milk, which further confirmed the formation of AgNPs in the presence of milk. The EDS profile of MP-s-AgNPs showed silver (Ag) and oxygen (O) peaks. The Ag peak was attributed to AgNPs and the O peak was attributed to the biomolecules in milk, which were bound to the surface of AgNPs, acting as capping and reducing agents. These biomolecules reduced Ag^+^ to Ag^0^ and stabilized the AgNPs, preventing their agglomeration for three months.

#### 3.1.7. Thermogravimetric Analysis (TGA) of the MP-s-AgNPs

**Figure 5:**
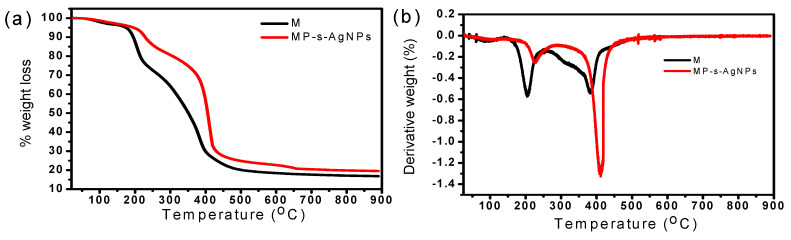
(**a**) TGA and (**b**) DTG curves of commercial milk (M) and MP-s-AgNPs.

The thermal stability of milk and the synthesized AgNPs were studied by TGA and differential thermogravimetry (DTG) and the results are given in Figure 5a,b, respectively. The DTG curve of milk exhibited two major peaks. The first peak at 206 °C corresponded to a significant weight loss (23%), which started from 142 °C and continued until 245 °C. Subsequently, at 381 °C, a complete weight loss of 44% was observed. The considerable weight loss until 421 °C was attributed to the degradation of protein constituents, that is, the breakdown of polypeptide chains of the whey protein and micelles of casein present in milk [92]. Normally in whey, proteins at a high-elevated temperature the large polypeptide changed to small polypeptide chains of amino acids [93] and strong casein micelles prevail to be dispersed [94].

On the other hand, for MP-s-AgNPs, weight reduction occurred in two stages. In the first stage, from 182 to 283 °C, 12% weight loss was observed, which was attributed to the degradation of proteins; the corresponding DTG peak was observed at 226 °C. While in the second stage (between 344 and 480 °C), 43% loss was observed, and the corresponding DTG peak was observed at 410 °C (Figure 5b). Thus, the thermal stability of MP-s-AgNPs was higher than that of milk, because of the presence of AgNPs.

### 3.2. Antimicrobial Activity of MP-s-AgNPs 

#### 3.2.1. Antibacterial Assay

Protein functionalization of metal nanoparticles (MNPs) can occur via electrostatic and non-specific adsorption of proteins, covalent interaction with cysteine residues or formation of amide bonds between proteins and organic molecules bound to the -NPs surface (protein conjugation). Moreover, NPs surface-functionalized with proteins exhibit several unique properties, making them suitable for several biological applications.

**Figure 6:**
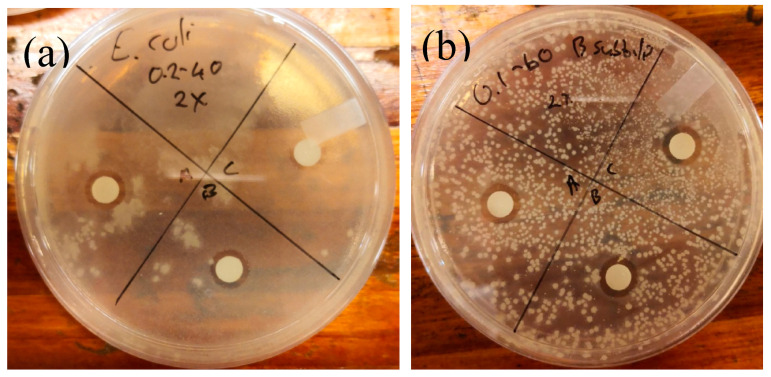
Photos showing disc diffusion test results: inhibition of (**a**) *Escherichia coli* and (**b**) *Bacillus subtilis*.

To evaluate the potential of MP-s-AgNPs, we conducted simple antimicrobial tests against selected bacteria. Disc diffusion assay was performed to determine whether the prepared MP-s-AgNPs could inhibit the growth of various bacteria. Inhibitory activity was observed for disc concentrations in the range of 12.5–800 µg/disc (Figure 6a,b).

Stronger inhibitory effects were observed at higher and lower concentrations of MP-s-AgNPs toward Gram (−ve) bacteria and Gram (+ve) bacteria, respectively. This result was attributed to the presence of an additional peptidoglycan layer in the Gram (−ve) cell, which served as an additional barrier toward the antimicrobial compound, limiting the effect of the antimicrobial compound. These results agreed with those of a previous study, wherein Gram (−ve) bacteria were found to be more resistant toward silver compounds than Gram (+ve) bacteria [95].

The positive control for Gram (−ve) cells (disc impregnated with carbenicillin) exhibited inhibition zones of 30.0 ± 1.41 mm (*S. typhi*) and 34.8 ± 1.26 mm (*E. coli*), while that for Gram (+ve) cells (disc impregnated with vancomycin) exhibited inhibition zones of 24.8 ± 1.26 mm (*B. subtilis*) and 18.3 ± 0.96 mm (*S. aureus*), as listed in Table 1. The mean values of inhibition zones generated by synthesized MP-s-AgNPs for various bacteria are listed in Table 2.

The MIC for *S. aureus* was determined to be 200 µg/L, verifying our observation that Gram (−ve) bacteria were more resistant to the antimicrobial compound because they exhibited higher colony-forming units per milliliter. This could be ascribed to the presence of the additional cell membrane that protected the cell contents, resulting in resistance to the inhibitory effect. The MBCs for *S. aureus* and *S. typhi* estimated by the broth dilution test were the same: 200 µg/mL. However, for both *E. coli* and *B. subtilis*, a higher MBC of 400 µg/mL was recorded. Surprisingly, the Gram (+ve) bacterium *B. subtilis* exhibited the same or higher resistance than Gram (−ve) bacteria toward the antimicrobial compound, implying that the inhibition observed in the first few hours against *B. subtilis* was mostly static; *B. subtilis*, a spore-forming bacterium, can go through a dormant phase to resist the inhibition. Otherwise, the antimicrobial compound would have swiftly acted to damage the cell membranes of the Gram (+ve) bacteria, resulting in perforation of the cell membranes and leakage of critical contents. This was due to the fact that the synthesized MP-s-AgNPs had large surface areas, which provided the bacterial cells with a large contact surface, resulting in the anchoring of particles onto the surface. Subsequently, the membrane perforated and the material entered the cell, forming a low-molecular weight region inside the cell. Although the bacteria conglomerated to protect the DNA structures, the nanoparticles attacked the cell respiratory chain, affecting the cell division, and thereby, resulting in cell death. A similar mechanism has been reported in the literature [96]. Furthermore, the AgNPs could interact with sulfur and phosphorous in the DNA, leading to signal transduction inhibition, and ultimately, cell lysis. The MIC and MBC for the various bacteria are presented in Table 3.

In some cases, the higher concentrations did not exhibit greater antimicrobial activity due to the specific mechanism of the compounds on the specific microorganism as the microorganism may anticipate the danger and develop resistance, especially at higher concentrations. This can explain the absence of the antibacterial effect at 800 µg/disc. To determine whether the compound induced a bactericidal or bacteriostatic effect, the MBC to MIC ratio was evaluated. In general, if the ratio is >4, the effect is considered bacteriostatic. However, in this study, the ratio did not exceed 4, therefore it was concluded that the effect was bactericidal, killing the bacteria and not only keeping it in the stationary growth phase.

Although the synthesized antimicrobial compound exhibited antibacterial activity, its performance was inferior to those of commercial antibiotics; nonetheless, a more competitive compound could be obtained via modification of the synthesis procedure. Furthermore, the advantage of protein capping is that it provides an anchoring layer, facilitating the transportation of drug or genetic materials to human cells [97]; moreover, the nontoxic protein cap could enhance the uptake and retention of the antimicrobial agent inside the human cells [98].

#### 3.2.2. Antifungal Assay

The results of the antifungal assay performed with the synthesized MP-s-AgNPs for the selected fungi are summarized in Table 4. Compared to the antifungal activity of the reference compound, AmB, the synthesized compound did not show significant inhibition. AmB produced inhibition zones of 7.88 ± 0.41, 13.25 ± 1.30 and 15.25 ± 1.92 mm for *A. fumigatus, A. ochraceus* and *P. chrysogenum*, respectively.

The minimum concentration of MP-s-AgNPs required for inhibition was 400 µg/disc, and with an increase in the concentration to 800 µg/disc, the inhibition slightly improved. The relatively high concentration required for the antifungal activity could be attributed to slow cell wall destruction. The longer time required for growth inhibition could be attributed to the adaptation of fungi to a hostile environment, resulting in the development of resistance to the MP-s-AgNPs, or destabilization of MP-s-AgNPs after a certain period under the experimental conditions. More satisfactory results could be possibly obtained when the AgNPs are delivered in combination with commercial compounds, providing a stronger inhibitory effect toward the fungi [99]. In addition, the results revealed that *A. ochraceus* and *P. chrysogenum* were the most susceptible toward the synthesized compound, while *A. fumigatus* showed higher resistance even at high concentrations, with a maximum MIZ of 0.03% ± 0.41% at 400 µg/disc. The low activity of the synthesized compound could be ascribed to the fact that the outer cell walls of *Aspergillus* spp. and *P. chrysogenum* were composed of hard chitin layers, protecting the fungal cell wall and the constituents, thereby requiring high levels of the antimicrobial compound to kill the fungi. Thus, the results showed that the synthesized MP-s-AgNPs were more harmful to bacteria than to fungi. 

## 4. Conclusions

In conclusion, we herein presented a method for the rapid, cost-effective, eco-friendly synthesis of non-toxic MP-s-AgNPs using milk. The synthesized AgNPs employing this method had good biocompatibility, enhanced colloidal stability and antibacterial activity. Highly stable AgNPs were obtained, with a fair control over their sizes, without using any stabilizing and reducing agent. The synthesized AgNPs were spherical and the elemental composition analysis confirmed the presence of silver and the absence of any contaminants. The prepared AgNPs exhibited significant antibacterial activity against common bacteria. Thus, these AgNPs could be potentially used for diverse applications in the fields of nano-biomaterials and antimicrobial therapy.

## Figures and Tables

**Table 1:** Mean values of inhibitory zones generated by the control compound for Gram (+ve) and Gram (−ve) bacteria.

Bacteria	Control Compound	Zone (mm)	Zone (mm)	Zone (mm)	Zone (mm)	Average Zone (mm)
*E. coli*	Carbenicillin	35	33	36	35	34.8 ± 1.26
*S. typhi*		30	31	28	31	30.0 ± 1.41
*S. aureus*	Vancomycin	18	17	19	19	18.3 ± 0.96
*B. subtilis*		25	26	23	25	24.8 ± 1.26

**Table 2:** Mean values and standard error of inhibitory zones generated by the antimicrobial compound for Gram (+ve) and Gram (−ve) bacteria.

	Average Zone (mm) ± Std Error			
Concentration (µg/disc)	*E. coli*	*S. typhi*	*B. subtilis*	*S. aureus*
800	14.8 ± 0.83	12.8 ± 0.83	11.8 ± 1.09	8 ± 0.71
400	9 ± 0.71	11.8 ± 0.56	13 ± 0.71	10.8 ± 1.92
200	8.5 ± 0.50	11.3 ± 0.83	11 ± 1.87	11.5 ± 0.93
100	8.5 ± 0.41	11 ± 0.35	8 ± 0.71	10.3 ± 0.83
50	8.3 ± 0.82	10.2 ± 0.89	9.5 ± 0.50	9.5 ± 0.79
25	7.5 ± 0.50	10.7 ± 0.65	7.5 ± 0.50	8.2 ± 0.89
12.5	7.3 ± 0.43	8.8 ± 0.56	10.3 ± 0.43	10.7 ± 1.19
6.25	7.3 ± 0.25	7.7 ± 0.41	7.5 ± 0.5	7.7 ± 0.41

**Table 3:** Minimum inhibitory concentration (MIC) and minimum bactericidal concentration (MBC) of the synthesized compound for various bacteria.

Selected Bacteria	MIC (µg/disc)	MBC (µg/mL)
*E. coli*	800	400
*S. typhi*	800	200
*B. subtilis*	400	400
*S. aureus*	200	200

**Table 4:** Minimum inhibition zones generated by the control (reference compound), AmB, for various strains of fungi.

Fungi	Control Compound	Zone (mm)	Zone (mm)	Zone (mm)	Zone (mm)	Average Zone (mm)
*A. fumigatus*	Amphotericin B	8	7.5	8.5	7.5	7.88 ± 0.41
*A. ochraceus*		14	12	15	12	13.25 ± 1.3
*P. chrysogenum*		12	16	17	16	15.25 ± 1.92

## Abbreviations

MP: milk protein; silver nanoparticles: AgNPs; UV-Vis: ultraviolet-visible; FTIR: Fourier-transform infrared, XRD: X-ray diffraction; SEM: scanning electron microscopy; TEM: transmission electron microscopy; DLS: dynamic light scattering; AmB: Amphotericin B; UHT: ultrahigh-temperature; MIZ: minimum inhibition zone; RT: room temperature; MBC: minimum bactericidal concentration; MIC: minimum inhibitory concentration; SAED: selected area electron diffraction; EDS: Energy-dispersive X-ray spectroscopy; DTG: differential thermogravimetry.
